# Seroprevalence and risk factors analysis of *Neospora caninum* and *Toxoplasma gondii* in cattle of Beheira, Egypt

**DOI:** 10.3389/fvets.2023.1122092

**Published:** 2023-02-13

**Authors:** Samy Metwally, Rania Hamada, Kamel Sobhy, Caroline F. Frey, Ragab M. Fereig

**Affiliations:** ^1^Infectious Disease Division, Department of Animal Medicine, Faculty of Veterinary Medicine, Damanhour University, Damanhour, Egypt; ^2^Clinical Pathology Division, Department of Pathology, Faculty of Veterinary Medicine, Damanhour University, Damanhour, Egypt; ^3^Department of Theriogenology, Faculty of Veterinary Medicine, Damanhour University, Damanhour, Egypt; ^4^Institute of Parasitology, Department of Infectious Diseases and Pathobiology, Vetsuisse-Faculty, University of Bern, Bern, Switzerland; ^5^Division of Internal Medicine, Department of Animal Medicine, Faculty of Veterinary Medicine, South Valley University, Qena, Egypt

**Keywords:** neosporosis, toxoplasmosis, seroprevalence, risk factors, cattle, Beheira, Egypt

## Abstract

**Introduction:**

*Neospora caninum* and *Toxoplasma gondii* are closely related obligate intracellular protozoan parasites. They are considered to be the major causes of infectious abortions and congenital abnormalities in livestock worldwide resulting in huge economic losses. Currently, there are no reports on the prevalence of neosporosis or toxoplasmosis in cattle in Beheira, Egypt's most important region for cattle industry.

**Methods:**

The current study investigated the presence of anti-*N. caninum* and anti-*T. gondii* antibodies in apparent healthy cattle from eight localities representing the whole area of Beheira. A total of 358 plasma samples were randomly collected from 6 dairy and 10 beef farms and analyzed by commercially available ELISAs. Production type (dairy versus beef), sex (female vs male), age (< 3 years, 3–5, and > 5 years old), breed (mixed vs Holstein vs Colombian Zebu), and location (various locations) were assessed as risk factors for *N. caninum* and *T. gondii* infections.

**Results and discussion:**

Of the samples, 88 (24.6%) and 19 (5.3%) were positive for anti-*N. caninum* and anti-*T. gondii* antibodies, respectively, and mixed infection was detected in 7. Of the 16 herds, 6 dairy and 7 beef herds were positive for antibodies to *N. caninum*. Antibodies to *T. gondii* were detected in 4, and 5 of dairy and beef herds, respectively. Production type (dairy) and, therewith, sex (female), age (aged over 5 years), and location were considered as risk factors for *N. caninum* infection. No factors statistically associated with *T. gondii* infection were identified. Overall, this study provided the first serological detection of *N. caninum* and *T. gondii* infections in cattle from Beheira, demonstrating the endemicity of both parasites in the main cattle rearing region of Egypt. This study also confirmed earlier reports of *N. caninum* being more present in dairy cattle than in beef cattle. Routine monitoring of *N. caninum* and *T. gondii* infections and the implementation of control strategies are urgently needed.

## Introduction

*Neospora caninum* and *Toxoplasma gondii* are single-celled, obligate intracellular protozoan parasites causing neosporosis and toxoplasmosis, respectively (1). They affect a variety of warm-blooded animals, including humans for *T. gondii*, and inflict serious clinical and economic losses, especially in the global cattle industry (2, 3). The reproductive stages of *T. gondii* are exclusively observed in members of the *Felidae* family (4), while canids serve as final hosts of *N. caninum* (5, 6). *Neospora caninum* is recognized as the most common infectious cause of abortion in cows globally (7). Early embryonic death and resorption, abortion, stillbirth, delivery of a calf with deformities, and the birth of healthy carrier offspring are all possible outcomes of *N. caninum* infection in pregnant cows (8). Early culling of seropositive cattle has been recorded (9) due to increased veterinary medical treatment expenditures and a drop in growth rates in these animals (10). Primary infection with *T. gondii* in cattle during gestation can cause abortion or congenital anomalies, including severe generalized toxoplasmosis with a possible fatal outcome (11). Infection in immunocompetent non-pregnant hosts, on the other hand, is frequently silent, resulting in mild or no clinical signs (12). The presence and spread of bovine neosporosis have been reported in more than 34 countries from Europe, Asia, America and Australia (13). However, few studies have investigated the prevalence of *N. caninum* infection in African countries (14). *Toxoplasma gondii* has been isolated from almost all geographical regions worldwide except Antarctica (15).

Egypt has a cattle population of about 5.1 million cattle (16) with the highest density in Beheira province. Beheira province is located in the Nile Delta, the northern (lower) part of Egypt. It embraces the whole of the delta west of the Rosetta branch with a considerable desert region to the south. Economically, agriculture is the most important sector in Beheira province and produces wheat, rice, and corn as the major crops. Other cereals, potatoes, sugar beets, tomatoes, and sesame are also harvested (17). The unique location of Beheira province made it an attractive area for investment in cattle production, and currently, it has the highest number of cattle farms in Egypt with approximately 20% of the total cattle population, i.e., about 1 Mio heads (16). The majority of cattle herds are either mixed native breeds, raised in small to medium-sized herds by local farmers, or imported cattle breeds from Europe, Africa, and South America with improved meat and milk production characteristics, including Holstein Friesian, Zebu, Brown Swiss, and Simmental (16, 18). Cattle farming systems are either intensive (more than 200 cattle), semi-intensive (20–200 cattle per farm), or smallholder (5–20 cattle per farm) systems (19).

Several investigations have been conducted in Egypt to determine the prevalence of neosporosis and toxoplasmosis in various animal species and people from different geographic locations (southern and northern) (3, 19–30). However, there has never been a thorough investigation of the prevalence of these parasites in cattle of the Beheira area. As reported in a literature review, only 15 blood samples from an abattoir in Beheira were analyzed in 1977 (31), and 7 (46.6%) of those samples proved positive for *T. gondii* antibodies. Therefore, this study aimed to investigate the presence of anti-*N. caninum* and anti-*T. gondii* antibodies in apparently healthy cattle from different localities and farms in Beheira province, and to provide insight into the risk factors related to such infections. We opted for commercial ELISAs in this study to obtain objective results that will allow for comparisons between different laboratories and countries.

## Materials and methods

### Ethical approval

This research has obtained the approval of the Ethics of the Institutional Committee of the Faculty of Veterinary Medicine at Damanhour University, Egypt (DMU/VetINF-2019-/0145). The project and procedures were explained to the animal owners, and verbal consent was obtained, as requested by the Ethics Committee.

### Sample population

**Figure 1:**
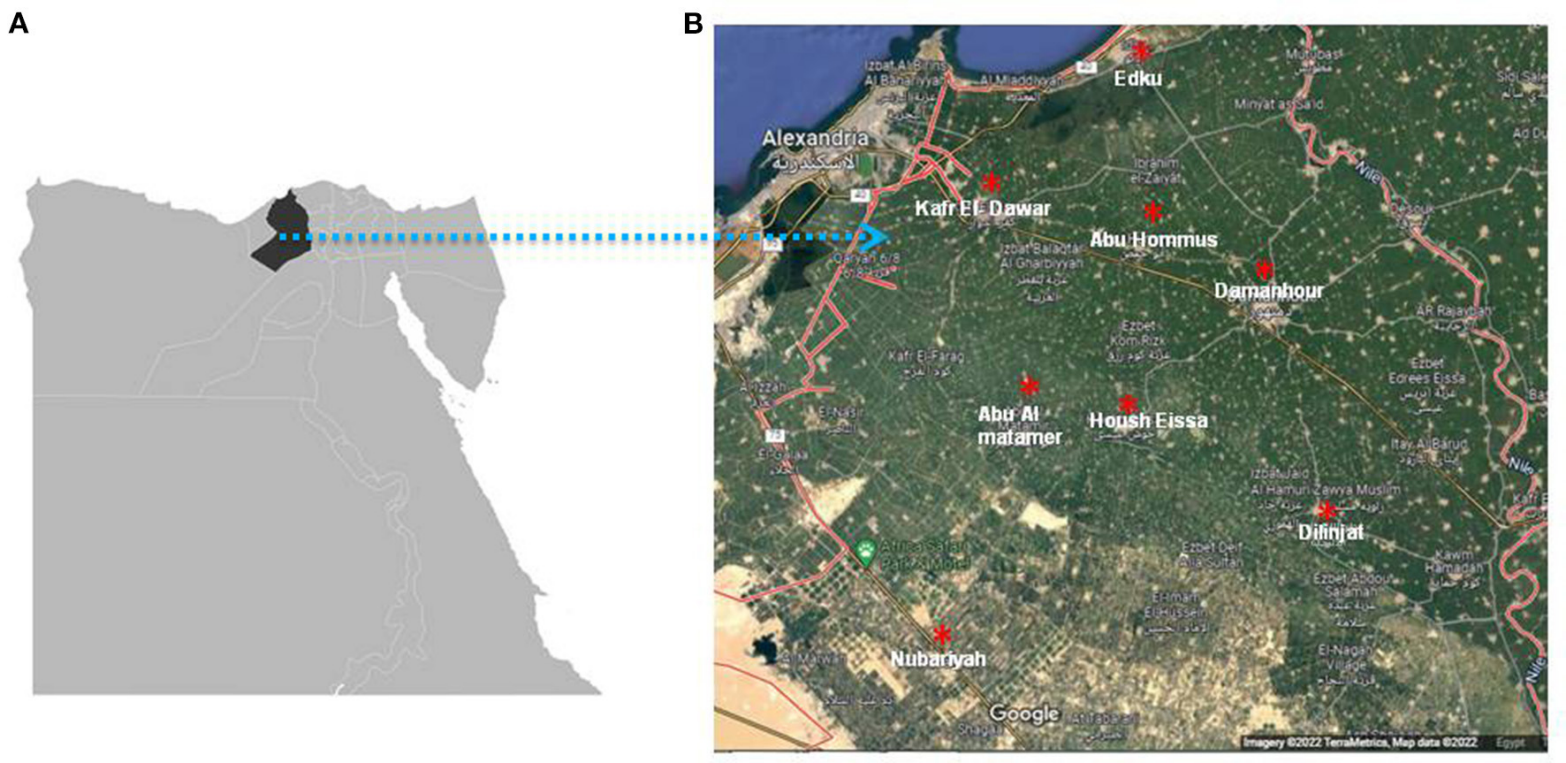
The geographical map of Egypt (Google imagery terraMetrics, Map data 2022) shows the location of Beheira province indicated by black color **(A)** and landscape showing the geographical distribution of eight localities in Beheira which indicated by red asterisks **(B)**.

A total number of 358 apparently healthy cattle of different ages: < 3 years (*n* = 142), 3–5 years (*n* = 143), and >5 years (*n* = 73); sexes: males (*n* = 142), and females (*n* = 216); production types: beef (*n* = 142), and dairy (*n* = 216); breeds: mixed (*n* = 205), Holstein (*n* = 109), and Colombian zebu (*n* = 44) were randomly selected and stratified according to cattle population size from 16 herds (6 dairy and 10 beef) comprising a total of 4,795 heads in 8 different localities (Table 1) namely: Damanhour (*n* = 20), Edku (*n* = 22), Abu Hommus (*n* = 78), Nubariyah (*n* = 56), Abu Almatamer (*n* = 27), Dilinjat (*n* = 60), Kafr El-Dawar (*n* = 72), and Housh Eissa (*n* = 23) in Beheira province, northern Egypt (Figure 1), during the first half of 2022. The number of the tested herds per locality varied between one and three (Table 3). About 10% of the animals of each farm were sampled (Table 1).

**Table 1:** Number and locations of tested samples of cattle from Beheira, Egypt.

**Locality**	Damanhour^*^	**Edku**	**Abu hommus**	**Nubariyah**	**Abu almatamer**	**Dilinjat**	**Kafr el-dawar**	**Housh eissa**	**Total**
**Population**									
No. of tested herds	2	1	3	1	1	3	3	2	16
No. of samples	20	22	78	56	27	60	72	23	358
Total no. of animals in tested herds	200	195	750	2,000	100	630	700	220	4,795

* Damanhour is the capital and biggest city of Beheira governorate.

### Blood sampling and plasma separation

Whole blood samples were collected from cattle using the tail vein puncture procedure and were stored in a glass tube with K_2_ EDTA anticoagulant. Blood samples were transported immediately to the laboratory and kept at 4°C. For serological investigation, plasma samples were separated by centrifugation at 3,000 rpm for 15 min at room temperature and kept at −20°C prior to analysis.

### Serological detection of *N. caninum* and *T. gondii* antibodies by ELISA

All 358 collected cattle plasma samples were serologically investigated for infection by *N. caninum* or *T. gondii* or mixed infection *via* indirect ELISA assay, using commercial ELISA kits. For *N. caninum*, samples were analyzed with the competitive multi-species ELISA for neosporosis (ID.vet, Grabels, France). Such test has the potential to detect IgG and IgM through the use of purified extract of *N. caninum* as coated antigen and anti-*N. caninum*-HRP, with recorded sensitivity (100%; CI 95%: 98.8–100%) and specificity (100%; CI 95%: 99.63–100%) as provided by the manufacturer. Plasma samples and controls were diluted 1:2. The ODs obtained were used to calculate the percentage of sample (*S*) to negative (*N*) ratio (S/N%) for each of the test samples according to the following formula S/N (%) = OD sample/OD negative control × 100. Samples with an S/N% greater than 60% were considered negative; if the S/N% was between 50 and 60%, the result was considered doubtful, and considered positive if the S/N% was less than 50%.

Regarding *T. gondii*, the samples were analyzed with the indirect multi-species ELISA for toxoplasmosis (ID.vet, Grabels, France) according to the manufacturers' instructions. This tool was specified to detect IgG through the use of P30 as coated antigen and anti-multispecies IgG-HRP, with recorded sensitivity (98.36%; CI 95%: 95.29–99.44%) and specificity (99.42%; CI 95%: 98.8–100%). Plasma samples and controls were diluted 1:10. The optical density (OD) obtained was used to calculate the percentage of sample (*S*) to positive (*P*) ratio (S/P%) for each of the test samples according to the following formula: S/P (%) = (OD sample–OD negative control)/(OD positive control–OD negative control) × 100. Samples with an S/P% less than 40% were considered negative; if the S/P% was between 40 and 50%, the result was considered doubtful, and considered positive if the S/P% was greater than 50%.

The ODs of all ELISA results were read at 450 nm and measured with an Infinite^®^ F50/Robotic ELISA reader (Tecan Group Ltd., Männedorf, Switzerland).

### Statistical analysis

The significance of the differences in the infection rates of the different diseases and risk factors was determined by the Fisher Exact Probability Test (two-tailed) using online statistics software http://vassarstats.net/ and GraphPad Prism version 5. A *P* value of < 0.05 was considered statistically significant. Odds ratios and the 95% confidence intervals were calculated using www.vassarstats.net and GraphPad Prism version 5.

## Results

Out of the 358 individual samples, 88 (24.6%) were positive for anti-*N. caninum* antibodies, 17 (4.7%) doubtful, and 253 (70.7%) were negative. A lower prevalence rate of anti-*T. gondii* antibodies was shown: 19 (5.3%) were positive, 22 (6.1%) doubtful, and 317 (88.5%) negative. Furthermore, 7 were positive and 3 were doubtful for both anti-*N. caninum* and anti-*T. gondii* antibodies (Table 2).

**Table 2:** Seroprevalence of *N. caninum, T. gondii*, and mixed infection in individual samples (*n* = 358).

**Type of infection**	**No. of negative (%)**	**No. of doubtful (%)**	No. of positive (%; 95% CI^*^)
*N. caninum*	253 (70.7)	17 (4.7)	88 (24.6; 20.3–29.4)
*T. gondii*	317 (88.5)	22 (6.1)	19 (5.3; 3.3–8.3)
Mixed infection	348 (97.2)	3 (0.8)	7 (2; 0.9–4.2)

^*^95% CI calculated according to method described by Available online at: http://vassarstats.net/ (accessed September 4–6, 2022).

On the herd level, 6/6 (100%) dairy and 7/10 (70%) beef herds tested positive for *N. caninum* antibodies, while *T. gondii* antibodies were detected in 4/6 (66.6%) dairy and 5/10 (50%) beef herds, respectively (Table 3). A higher seroprevalence of *N. caninum* was reported in dairy herds of mixed breeds with percentages of 54.6, 50, 34.8, and 11.1% in herds # 3, 12, 9, and 8, respectively, than in herds of Holstein cows (27.5, and 10.7% in herds # 4, and 7, respectively). Among beef herds, Colombian zebu herds # 11, and 2 showed the highest seroprevalence of *N. caninum*, with percentages of 60 and 40%, respectively, however zebu herds # 14 and 16 were totally negative. Herds with both, mixed and Holstein beef cattle, showed seroprevalences of 5, 16.7, 29.6, 20, and 7.1% in herds # 5, 6, 10, 13, and 15, respectively, however herd # 1 tested negative (Table 3).

**Table 3:** Herd level seroprevalence of *N. caninum, T. gondii*, and mixed infection.

**Herd**	**Region**	**Sex**	**Breed**	**Production type**	**No. of tested animals in herd**	***N. caninum***		***T. gondii***		**Mixed infection**	
						**No. of positive individuals (%; 95% CI** ^*^ **)**	**Herd serostatus**	**No. of positive individuals (%; 95% CI** ^*^ **)**	**Herd serostatus**	**No. of positive individuals (%; 95% CI** ^*^ **)**	**Herd serostatus**
#1	Damanhour	Male	Mixed	Beef	15	0 (0; 0–25.4)	Negative	2 (13.3; 2.3–41.6)	Positive	0	Negative
#2	Damanhour	Male	Colombian Zebu	Beef	5	2 (40; 7.3–83)	Positive	0 (0; 0–53.7)	Negative	0	Negative
#3	Edku	Female	Mixed	Dairy	22	12 (54.6; 32.7–74.9)	Positive	2 (9; 1.6–30.6))	Positive	0	Negative
#4	Abu Hommus	Female	Holstein	Dairy	40	11 (27.5; 15.1–44.1)	Positive	0 (0; 0–10.9)	Negative	0	Negative
#5	Abu Hommus	Male	Mixed & Colombian Zebu	Beef	20	1 (5; 0.3–26.9)	Positive	1 (5; 0.3–26.9)	Positive	0	Negative
#6	Abu Hommus	Male	Mixed & Colombian Zebu	Beef	18	3 (16.7; 4.4–42.3)	Positive	0 (0; 0–21.9)	Negative	0	Negative
#7	Nubariyah	Female	Holstein	Dairy	56	6 (10.7; 4.4–22.6)	Positive	0 (0; 0–8)	Negative	0	Negative
#8	Abu Almatamer	Female	Mixed	Dairy	27	3 (11.1; 2.9–30.3)	Positive	3 (11.1; 2.9–30.3)	Positive	1 (3.7; 0.2–20.9)	Positive
#9	Dilinjat	Female	Mixed	Dairy	23	8 (34.8; 17.2–57.2)	Positive	1 (4.3; 0.2–24)	Positive	1 (4.3; 0.2–24)	Positive
#10	Dilinjat	Male	Holstein & Mixed	Beef	27	8 (29.6;14.5–50.3)	Positive	0 (0; 0–15.5)	Negative	0	Negative
#11	Dilinjat	Male	Colombian Zebu	Beef	10	6 (60; 27.4–86.3)	Positive	0 (0; 0–34.5)	Negative	0	Negative
#12	Kafr El–Dawar	Female	Mixed	Dairy	48	24 (50; 35.4–64.6)	Positive	7 (14.6; 6.5–28.4)	Positive	5 (10.4; 3.9–23.5)	Positive
#13	Kafr El–Dawar	Male	Mixed	Beef	15	3 (20; 5.3–48.6)	Positive	1 (6.7; 0.4–34)	Positive	0	Negative
#14	Kafr El–Dawar	Male	Colombian Zebu	Beef	9	0 (0; 0–37.1)	Negative	1 (11.1; 0.6–49.3)	Positive	0	Negative
#15	Housh Eissa	Male	Mixed	Beef	14	1 (7.1; 0.4–35.8)	Positive	1 (7.1; 0.4–35.8)	Positive	0	Negative
#16	Housh Eissa	Male	Colombian Zebu	Beef	9	0 (0; 0–37.1)	Negative	0 (0; 0–37.1)	Negative	0	Negative
Total	8	2	3	2	358	88 (24.6; 20.3–29.4)	13 of 16 positive	19 (5.3; 3.3–8.3)	9 of 16 positive	7 (7.1; 0.9–4.2)	3 of 16 positive

^*^95% CI calculated according to the method described by Available online at: http://vassarstats.net/ (accessed September 4–6, 2022).

Regarding *T. gondii* seroprevalence in dairy herds, positive cases were reported only in mixed breed herds with percentages of 9, 11.1, 4.3, and 14.6% in herds # 3, 8, 9, and 12, respectively. Also for seropositive beef herds, 4 herds were mixed cattle herds with infection rates of 13.3, 5, 6.7, and 7.1% in herds # 1, 5, 13, and 15, respectively, and one was a Colombian zebu herd (#14) with 11.1% infected animals. Co-infection of the same animal with *N. caninum* and *T. gondii* was detected only in dairy herds # 8, 9, and 12, all with mixed cattle breed, with co-infection rates of 3.7, 4.3, and 10.4%, respectively (Table 3).

The impact of several risk factors such as age, sex and production type, breed, and location of the tested cattle on the seroprevalence of *N. caninum* and *T. gondii* was investigated. For *N. caninum* infection, prevalence in cattle < 3 years old was 24/142 (16.9%), and a non-significant increase [*p* = 0.23; odds ratio (OR) = 1.5] in seropositivity was observed in cattle aged 3–5 years (33/143; 23.1%). A significant increase in *N. caninum* seroprevalence (*p* < 0.0001; OR = 3.6) was demonstrated in older cattle aged >5 years (31/73, 42.5%; Table 4). Female cattle (dairy cows) showed a significantly higher seroprevalence for *N. caninum* (64/216, 29.6%; *p* = 0.008, OR = 2.1) than males that were used for beef production (24/142, 16.9%) (Table 4). Only non-significant differences between different breeds were found. The lowest prevalence of *N. caninum* infection was detected in Colombian zebu (18.2%). Holstein cattle showed a higher seroprevalence (21.1%), and mixed cattle had the highest infection rate (27.8%) (Table 4). Seroprevalence for *N. caninum* varied between the localities. The prevalence in Damanhour (2/20; 10%) was taken as a reference value, and thus cattle in Edku (12/22; 54.5%), Kafr El-Dawar (27/72; 37.5%), and Dilinjat (22/60; 36.7%) had significantly higher values in comparison (*p* = 0.003, 0.027; 0.026; ORs = 10.8, 5.4, 5.2, respectively) (Table 4). No significant differences compared to Damanhour were found in Abu Hommus (15/78; 19.2%), Abu Almatamer (3/27; 11.1%), Nubariyah (6/56; 10.7%), and Housh Eissa (1/23; 4.3%) (Table 4).

**Table 4:** Risk factors for *N. caninum* infection in cattle in Beheira, Egypt.

**Analyzed factor**	**No. of Tested**	**No. of negative (%)**	**No. of positive (%)**	**OR (95% CI)^#^**	*P-*value^*^
**Age**					
< 3 years	142	118 (83.1)	24 (16.9)	Ref	Ref
3–5 years	143	110 (76.9)	33 (23.1)	1.5 (0.8–2.7)	0.23
>5 years	73	42 (57.5)	31 (42.5)	3.6 (1.9–6.9)	0.0001
**Sex**					
Male	142	118 (83.1)	24 (16.9)	Ref	Ref
Female	216	152 (70.4)	64 (29.6)	2.1 (1.2–3.5)	0.008
**Production type**					
Beef	142	118 (83.1)	24 (16.9)	Ref	Ref
Dairy	216	152 (70.4)	64 (29.6)	2.1 (1.2–3.5)	0.008
**Breed**					
Mixed	205	148 (72.2)	57 (27.8)	1.7 (0.8–4)	0.25
Holstein	109	86 (78.9)	23 (21.1)	1.2 (0.5–2.9)	0.82
Colombian zebu	44	36 (81.8)	8 (18.2)	Ref	Ref
**Location**					
Damanhour	20	18 (90)	2 (10)	Ref	Ref
Edku	22	10 (45.5)	12 (54.5)	10.8 (2–85.3)	0.003
Abu Hommus	78	63 (80.8)	15 (19.2)	2.1 (0.4–10.3)	0.51
Nubariyah	56	50 (89.3)	6 (10.7)	1.1 (0.2–5.8)	1
Abu Almatamer	27	24 (88.9)	3 (11.1)	1.1 (0.2–7.5)	1
Dilinjat	60	38 (63.3)	22 (36.7)	5.2 (1.1–24.6)	0.026
Kafr El-Dawar	72	45 (62.5)	27 (37.5)	5.4 (1.2–25)	0.027
Housh Eissa	23	22 (95.7)	1 (4.3)	0.4 (0.03–4.9)	0.6

^#^Odds ratio at 95% confidence interval as calculated by Available online at: http://vassarstats.net/. ^*^P value was evaluated by Fisher Exact Probability Test (two-tailed) using online statistics software http://vassarstats.net/ and GraphPad Prism version 5. The result is significant at *P* < 0.05. Ref., value used as reference.

For *T. gondii*, none of the tested factors (age, sex and production type, breed, and locality) had a significant effect on the seroprevalence. The lowest infection rate was found in cattle 3–5 years old (3.5%) followed by the youngest group (< 3 years; 4.2%). A slight, non-significant increase was found in the oldest group (>5 years; 11%) (*p* = 0.15, OR = 2.8) (Table 5). The infection rate with *T. gondii* in males was lower than in females, but also non-significantly (4.2 vs. 6%) (Table 5). Seroprevalence rates in the differed breeds varied from 0% in Holstein cattle, 2.3% in Colombian zebu cattle, up to 8.8% in mixed cattle breed, but there were no statistical differences (Table 5). Regarding location, cattle samples from Kafr El-Dawar showed the highest seroprevalence for *T. gondii* (12.5%) followed by Abu Almatamer (11.1%), Damanhour (10%), Edku (9.1%), Hosh Eissa (4.3%), Dilinjat (1.7%), and Abu Hommus (1.3%), but no significant differences between these regions were found (Table 5). Noticeably, a complete absence of *T. gondii* infection was reported in tested cattle from Nubariyah but there was no significant difference to Damanhour (Table 5).

**Table 5:** Risk factors for *T. gondii* infection in cattle in Beheira, Egypt.

**Analyzed factor**	**No. of Tested**	**No. of negative (%)**	**No. of positive (%)**	**OR (95% CI)^#^**	*P*–value^*^
**Age**					
< 3 years	142	136 (95.8)	6 (4.2)	Ref	Ref
3–5 years	143	138 (96.5)	5 (3.5)	0.8 (0.2–2.8)	0.77
>5 years	73	65 (89)	8 (11)	2.8 (0.9–8.4)	0.15
**Sex**					
Male	142	136 (95.8)	6 (4.2)	Ref	Ref
Female	216	203 (94)	13 (6)	1.5 (0.5–3.9)	0.63
**Production type**					
Beef	142	136 (95.8)	6 (4.2)	Ref	Ref
Dairy	216	203 (94)	13 (6)	1.5 (0.5–3.9)	0.63
**Breed**					
Mixed	205	187 (91.2)	18 (8.8)	4.1 (0.5–31.9)	0.21
Holstein	109	109 (100)	0	0.1 (0.005–3.3)	0.28
Colombian zebu	44	43 (97.7)	1 (2.3)	Ref	Ref
**Location**					
Damanhour	20	18 (90)	2 (10)	Ref	Ref
Edku	22	20 (90.9)	2 (9.1)	0.9 (0.1–7.1)	1
Abu Hommus	78	77 (98.7)	1 (1.3)	0.1 (0.01–1.4)	0.1
Nubariyah	56	56 (100)	0	0.06 (0.003–1.4)	0.06
Abu Almatamer	27	24 (88.9)	3 (11.1)	1.1 (0.2–7.5)	1
Dilinjat	60	59 (98.3)	1 (1.7)	0.2 (0.01–1.8)	0.15
Kafr El–Dawar	72	63 (87.5)	9 (12.5)	1.3 (0.3–6.5)	1
Housh Eissa	23	22 (95.7)	1 (4.3)	0.4 (0.03–4.9)	0.59

^#^Odds ratio at 95% confidence interval as calculated by Available online at: http://vassarstats.net/. ^*^P value was evaluated by Fisher Exact Probability Test (two-tailed) using online statistics software http://vassarstats.net/ and GraphPad Prism version 5. The result is significant at *P* < 0.05. Ref., value used as reference.

## Discussion

This study provided the first report on the seroprevalence of *N. caninum* and *T. gondii* in cattle from Beheira province as well as a respective risk factor analysis (age, sex, and therewith production type, breed, and location). Our findings indicated a greater individual infection rate with *N. caninum* (24.6%) than with *T. gondii* (5.3%), which was also reflected on the herd level with 13 herds positive for *N. caninum* compared to 9 herds for *T. gondii*. Reportedly, cattle are naturally more susceptible to *N. caninum* than *T. gondii* infection and *N. caninum* can persist in a herd due to the highly efficient vertical transmission from cow to calf (32, 33). Indeed, in endemically infected dairy cattle herds up to 95% of calves of seropositive cows are born infected (13, 34). In our study, the seroprevalence in the youngest age group (< 3 years) was almost 17%, very close to the one reported before for this age group (17.5%) by Fereig et al. (24). Furthermore, owners frequently use domestic dogs as guardian dogs in cattle farms, thus the final host for *N. caninum* is often in close contact with livestock. In addition, total numbers of dogs have increased significantly in recent years (35), and a large number of stray canines and wolves are present in Egypt (22, 36). Thus, environmental contamination with *N. caninum* oocysts is potentially high in Egypt, especially on cattle farms. The observed increase of seroprevalence for *N. caninum* with age in our study suggests horizontal infection with oocysts as important route of infection, in addition to vertical transmission.

In comparison to the previous studies on *N. canimum* in cattle in Egypt, this work reported a similar prevalence rate (24.6%) to the 20.43% reported in cattle of the Delta region (23), a neighboring region of Beheira governorate, and also to the 18.9% in cattle from southern Egypt (24). Recently, three of ten bulk milks from different farms and different regions in Egypt were shown to contain antibodies to *N. caninum*, and one of these bulk milks was additionally positive for *N. caninum* DNA (26). For other livestock like camels, variable seroprevalences of 3.6% (37), 11% (21), and 25.7% (25) have been reported. A seroprevalence of even 68% was found in water buffalo (1). Our findings for *T. gondii* showed a lower infection rate (5.3%) compared to earlier research that estimated the seroprevalence of anti-*T. gondii* antibodies in cattle in Egypt. For instance, using a TgSAG2t-ELISA, 10.8% of the cattle sera from the Delta region (23), and 23.6% of the cattle from Qena, Kafr Elsheikh, and Minoufia tested positive (19). In Shebein El-Kom, molecular detection in cattle milk revealed 19.35% (6/31) positive for *T. gondii* DNA (30), while one in ten bulk milks tested positive for *T. gondii* DNA in a recent study (26). Cattle meat samples from Sohag city showed 31.3% positive by PCR (20). Despite the fact that the area of investigation differ between the studies, no earlier studies have revealed a lower prevalence of *T. gondii* infection in cattle than ours. As cats are the primary source of infection with this protozoan, this could be a sign of improved cat control on cattle farms in Beheira. Indeed, the majority of cats in Beheira are now domesticated, and the number of stray cats has dropped considerably in past years. As an outcome, a progressive reduction in the number of new cattle infections was anticipated.

The positive rate in female dairy cattle (29.6%) was significantly higher than in male beef cattle (16.9%). This supports earlier observations by Quintanilla-Gozalo et al. (38), who recorded higher seroprevalences to *N. caninum* in dairy cattle (83.2 and 36.8%) than those in beef cattle (55.1 and 17.9%) at the herd and individual level, respectively. Age might be an important factor in this equation, as in our study most of the males were younger than 3 years old and females were >3 years. This might also explain the contrast with an earlier study that did not identify sex as a risk factor for infection with *N. caninum* (24). Therefore, further investigation into the relationship between age, sex, and production type in cattle is necessary to determine the exact contribution of each variable to the infection risk. In accordance with an earlier study (19), the breed of cattle had no impact on the prevalence of *N. caninum* infection. However, a trend toward higher infection rates for both, *N. caninum* and *T. gondii*, was seen in mixed breed herds when compared to Holstein and Colombian zebu cattle in our study. Moreover, co-infection was reported only in 3 dairy mixed herds. These data could be linked to the management of these herds. Most of mixed cattle herds were not completely fenced against stray animals, suffered from shortage in veterinary services and regular screening of diseases, and were restocking with their own calves. The importance of the management system on the prevalence rate of *N. caninum* was also reflected by the significantly higher seroprevalence in Edku (only mixed breed analyzed), Dilinjat (all three breeds), and Kafr El-Dawar (mixed breed and zebu), respectively. This could be attributed to the nature of farming systems and the density of farms in these localities where most of farms are semi-intensive in comparison to the well-controlled intensive farms in other localities of the sampling area. These intensive herds mainly kept foreign breeds and the management was more professional. Furthermore, most of these cattle were imported as certified-free from diseases prior to stocking. Biosecurity, good veterinary practices, and use of artificial insemination in breeding system were also advantageous (16, 18). Neither age, sex and production type, nor breed were identified as risk factors for *T. gondii* infection in the examined cattle. This result was consistent with an investigation of the risk variables linked to *T. gondii* infection in cattle in southern Egypt (19). It's important to note that no Holstein cattle tested positive for anti-*T. gondii* antibodies, most probably for the management reasons discussed above.

## Conclusion and recommendations

In conclusion, this study provided the first serological survey on subclinical infection with *N. caninum* and *T. gondii* in cattle in Beheira province. The findings indicated a higher prevalence of *N. caninum* compared to *T. gondii* among the cattle tested, as well as in comparison to earlier investigations carried out in Egypt. The most important risk factors for *N. caninum* infection in the cattle studied were age, sex, and therewith production type, and locality. However, the current investigation found no risk factors for *T. gondii* infection. Future research is needed to provide more detailed information about the prevalence of *N. caninum* and *T. gondii* in Egypt. The elaboration of effective control strategies and biosecurity measures in cattle farms has become an urgent need, because no vaccines or effective drugs against *N. caninum* or *T. gondii* are currently available.

## Data availability statement

The raw data supporting the conclusions of this article will be made available by the authors, without undue reservation.

## Ethics statement

This research has obtained the approval of the Ethics of the Institutional Committee of the Faculty of Veterinary Medicine at Damanhour University, Egypt (DMU/VetINF-2019-/0145). Written informed consent for participation was not obtained from the owners because oral consent was obtained from each owner, as was agreed upon by the Ethics Committee (see respective letter). In Egypt, written informed consent is very hard to obtain because verbal explanations and consequent consent is the usual way of reaching agreements.

## Author contributions

Conceptualization, design, resources, and shared materials: SM, RF, and CF. Experiments, formal analysis, and investigation: SM, RF, RH, KS, and CF. Writing—original draft: SM, RF, and RH. Writing—review and editing, project administration, and funding acquisition: RF and CF. All authors have read and agreed to the published version of the manuscript.
